# Change in atmospheric deposition during last half century and its impact on lichen community structure in Eastern Himalaya

**DOI:** 10.1038/srep30838

**Published:** 2016-08-09

**Authors:** Rajesh Bajpai, Seema Mishra, Sanjay Dwivedi, Dalip Kumar Upreti

**Affiliations:** 1 Lichenology Laboratory, Plant Diversity Systematics and Herbarium Division, Lucknow, India; 2 Plant Ecology and Environment Science Division, CSIR-National Botanical Research Institute, Rana Pratap Marg, Lucknow - 226001, India

## Abstract

Climatic fluctuations largely affects species turnover and cause major shifts of terrestrial ecosystem. In the present study the five decade old herbarium specimens of lichens were compared with recent collection from Darjeeling district with respect to elements, PAHs accumulation and carbon isotope composition (δ^13^C) to explore the changes in climatic conditions and its impact on lichen flora. The δ^13^C has increased in recent specimens which is in contrast to the assumption that anthropogenic emission leads to δ^13^C depletion in air and increased carbon discrimination in flora. Study clearly demonstrated an increase in anthropogenic pollution and drastic decrease in precipitation while temperature showed abrupt changes during the past five decades resulting in significant change in lichen community structure. The Usneoid and Pertusorioid communities increased, while Physcioid and Cyanophycean decreased, drastically. Lobarian abolished from the study area, however, Calcicoid has been introduced in the recent past. Probably, post-industrial revolution, the abrupt changes in the environment has influenced CO_2_ diffusion and/C fixation of (lower) plants either as an adaptation strategy or due to toxicity of pollutants. Thus, the short term studies (≤5 decades) might reflect recent micro-environmental condition and lichen community structure can be used as model to study the global climate change.

The climate change due to pollution appears to be one of the serious global threats expected in the foreseeable future. The global average temperature has increased by approximately 0.8 °C during last 5–6 decades. Combustion of fossil fuels, emissions of halocarbons and other green-house gases, deforestation, land-cover change has contributed in global warming^1^,^2^,^3^,^4^. A drastic increase in CO_2_ concentration and change in isotopic composition of atmospheric carbon dioxide (δ^13^C) has been observed during second half of the last century^1^,^5^. Climatic alterations not only affects natural ecosystem but each and every species and communities on the earth is being affected to a lesser or greater extent^6^,^7^. Shrinking and shifting of habitats, change in communities, extinction of species and physiological and behavioural changes in biota has been observed as an impact of global climate change^8^,^9^. The consequences of global climate change have awakened most of the countries to pay attention on reliable techniques to forecast the climate changes and to evaluate its effects on flora and fauna^10^,^11^,^12^,^13^. The evaluation of climatic changes is generally monitored by physico-chemical detectors, which provide quantitative data on air, water and soils; conversely, the biological monitoring is a potential tool for assessing environmental pollution and its impact on biological variables even up to centuries back^14^. Such studies include communities and species composition exposed to different kinds of pollution and its comparison with historical data of decades to centuries present as herbarium records^15^,^16^,^17^. Primack *et al.*^18^ demonstrated that herbarium specimens collected over many years could be combined with a single baseline season of field observations to provide a source of data for changes in plant flowering time.

Lichens, a symbiotic association between a fungus and an alga, colonize 8% of the terrestrial surface of the earth. The peculiar symbiotic association enables lichens to colonize on diverse range of habitats such as temperate and tropical regions, hot to dry deserts and arctic tundra. They can even survive in space exposed to extraterrestrial solar UV and cosmic radiation^19^. The lack of vascular system and dependence to absorb water and nutrients passively from their environment make lichens sensitive to environmental factors such as temperature, water availability and air pollutants^20^,^21^. Since the growth of various lichen species is heavily dependent on the climate, a minor fluctuation in the climate may change the community structure^22^,^23^. Lichen community composition disturbance can provide information about alteration in climatic conditions and air quality of the area^24^,^25^,^26^. Thus, shift in lichen distribution and its use as indicators of air pollutants has been well studied in European countries, northern America and South-east Asia^27^,^28^,^29^,^30^,^31^,^32^. During the last decades several studies have used herbarium lichen specimens as a tool for determining the early twentieth century environmental conditions to compare with present atmospheric pollution^26^,^33^. For instance, Isocrono *et al.*^34^ found a change in lichen diversity over a period of 200 years in the city of Turin, north Italy, with higher abundance of lichen species in 19^th^ century to a drastic decrease between 1960 and 1996 and further reappearance in 1999 related to change in air quality in the city centre. Root *et al.*^35^ suggested that different lichen species can be useful for monitoring different trends in climate such as *Hypogymnia apinnata* and *Bryoria glabra* are indicator of sub oceanic climate while *Alectoria sarmentosa*, *Plastismatia norvegica* were typical for oceanic climate. The carbon isotope composition (δ^13^C) of herbarium samples has also been used as a tool to represent changes in atmospheric CO_2_ concentration and isotopic composition related to anthropogenic activity^36^,^37^. Zschau *et al.*^38^ correlated the atmospheric deposition of trace elements on lichen genus *Xanthoparmelia* with specimens preserve in the herbaria and concluded that the trace elements were increased in Arizona due to various anthropogenic activities. Purvis *et al.*^33^ compared herbarium samples with respect to elements signatures to reconstruct the historical trends in atmospheric deposition and changing pollution sources. In the view of above, it may be assumed that metals concentrations in the herbarium lichen samples correlate with atmospheric inputs for the corresponding period, thus herbarium specimens can be safely used in environmental studies provided the disruptive factors such as sampling contamination, preservatives and storage condition can be excluded^39^.

Himalayan region is particularly characterized for a rich biodiversity including medicinal, bioprospecting and indicator species. According to IPCC (Intergovernmental Panel on Climate Change) projection Himalayas may suffer drastic climate changes. The rapid temperature increase and changes in precipitation, in combination with the importance of Himalayan snowpack and glaciers, make the region one of the most threatened nonpolar areas of the world^40^,^41^. Rapid shrinking of Himalayan glaciers has been observed which is more drastic in eastern region of Himalaya^42^. Darjeeling, situated in foothills of the Eastern Himalayas in India having significant altitudinal variation, from 130 to 3660 m, exhibit a wide array of agro-climatic zones, which favour the luxuriant growth of diversified and rich vegetation including lichens^43^. In the present study, we investigated the changes in atmospheric deposition, in terms of elemental composition and PAHs accumulation, as well as in δ^13^C as a representative of global CO_2_ increase and the impact on lichen community structure to study the global climate/microclimate change during last half century using herbarium specimens.

## Materials and Methods

### Study area and sample collection

CSIR-National Botanical Research Institute, herbarium (LWG) is housing rich collection of lichen specimens representing almost all the phyto-geographical regions of the country. Among the various Himalayan regions, the Darjeeling district situated in eastern Himalaya is well explored for its lichens and a large number of identified specimens are preserved in the herbarium. The herbarium was investigated to find out the old herbarium records of lichens from Darjeeling district (87°59′–88°53′E and 28°31′–27°13′N) in the state of West Bengal. The herbarium specimen of lichens used in the present study was collected by late Professor D. D. Awasthi and his group in the year 1966 from 11 localities, representing most of the area of Darjeeling district^43^,^44^ (Supplementary Table 1). After preparing the check list of lichens and details of localities, the area was revisited in the year 2014 to study current lichen diversity and to collect fresh samples for the analysis of various parameters.

### Climatic condition of the area

The daily meteorological data (temperature, precipitation and humidity) of Darjeeling for the whole study duration was obtained from Indian Meteorology Department (IMD), Pune, Government of India. The annual mean of each meteorological parameter was calculated from the daily record covering the period of 1966–2015.

### Analysis of organic and inorganic pollutants

After gone through the herbarium records, it has been found that *Heterodermia diademata* (Taylor) D. D. Awasthi, was the common foliose lichen growing luxuriantly in 1966 at all eleven sites, and also encountered in the fresh survey. Therefore, the *H. diademata* was selected to analyze the level of organic (PAHs), inorganic (Fe, Zn, Co, Ni, Cu, Se, Mn and As, Cr, Pb) pollutants and climate change related parameters (carbon, nitrogen content and carbon isotope composition). At least three lichen samples with 3 replicate each (n = 9) from each site was use for the analysis of various parameters. The details of methodology followed for each parameter is as under:

#### Polycyclic aromatic hydrocarbons (PAHs) estimation

The estimation of PAHs was performed according to the procedure of Environmental Protection Agency -EPA 8310 (US EPA 1986^45^). Lichen samples (1.0 g) were extracted in 100 ml of Dichloromethane (Merck, AR) for 16 hours using a Soxhlet apparatus. The extract was passed through anhydrous sodium sulphate (Qualigen, AR) to remove moisture and then concentrated to 2 ml under vacuum in Buchi rotary evaporator. The extract was purified on a silica gel (100/200 mesh size, Qualigen) column using hexane according to the EPA method 3630. The purified extract was solvent exchanged to acetonitrile (Merck, AR) and final volume was made to 2 ml in umber coloured volumetric flask. Samples were stored in dark at 4 °C till the analysis of PAHs.

The PAHs were separated using reverse phase C-18 column (250 nm × 4.6 mm, 5 μm particle size; Waters) on a HPLC consisting 515 pump (Waters milford, MA, USA) and UV–visible detector (2487, Waters). The PAHs were eluted through 70% (v/v) acetonitrile at flow rate of 1.5 ml/min at 27 °C. The chromatogram was recorded at 254 nm and processed using the software Empower^TM^. The identification and quantification was performed by using the respective PAH standards procured from Sulpelco, USA. The limit of detection for individual PAHs ranged between 10–30 ng g^−1^.

### Elemental analysis

The oven-dried (70 °C) lichen samples were grounded to fine powder and digested (0.5 g) in HNO_3_:H_2_O_2_ (3:1 v/v). After digestion the volume was made to 5 ml by Milli Q water. Prior to analysis the samples were diluted 10 times and the concentration of elements (Fe, Zn, Co, Ni, Cu, Se, Mn and As, Cr, Pb) was analysed using an Inductively Coupled Plasma Mass Spectrometer (ICP-MS, Agilent 7500 ce) as detailed in Dwivedi *et al.*^46^. Rhodium (4 μg l^−1^) was added to all samples for internal standardization.

The standard reference materials of metals/metalloids (E-Merck, Germany) were used for the calibration and quality assurance for each analytical batch. Analytical data quality of metals/metalloids was ensured with repeated analysis (n = 5) of quality control samples, and the results were found within (±2.82) the certified values. Recovery of Fe, Zn, Mn, Cu, Co, Se, Cr, Pb and As from the samples were found to be more than 98%, as determined by spiking of samples with a known amount of elements. The detection limit for each element was 1 μg l^−1^.

### Estimation of carbon, nitrogen content and carbon isotope composition

The carbon and nitrogen concentration of lichen samples were analysed by an elemental analyser (EA 1108, Carlo-Erba-Milano, Italy) with an analytical precision of 0.1%. The stable C isotopic ratio was measured with an isotope ratio mass spectrometer (CONFLO interface, Thermo, MAT Bermen, Germany) operating in continuous flow mode after the combustion of the samples in an elemental analyser (EA 1108, Carlo-Erba-Milano, Italy). Samples were weighted by using a high precision Ultra Micro Balance and the percentage composition were calculated based on Carlo Erba Elemental Standards B2005, B2035, and B2036, with an error of <1%. Standards of ammonium sulphate (IAEA-N1 and IAEA-N2) for nitrogen, and sugar (IAEA-CH6) and graphite (EIL-32) for carbon were used for calibration.

### Statistical analysis

Two-way analysis of variance (ANOVA) and Duncan’s multiple range test (DMRT) were performed between the different parameters by fallowing Gomez and Gomez^47^.

## Results and Discussion

### Change in climatic condition of the area

**Figure 1:**
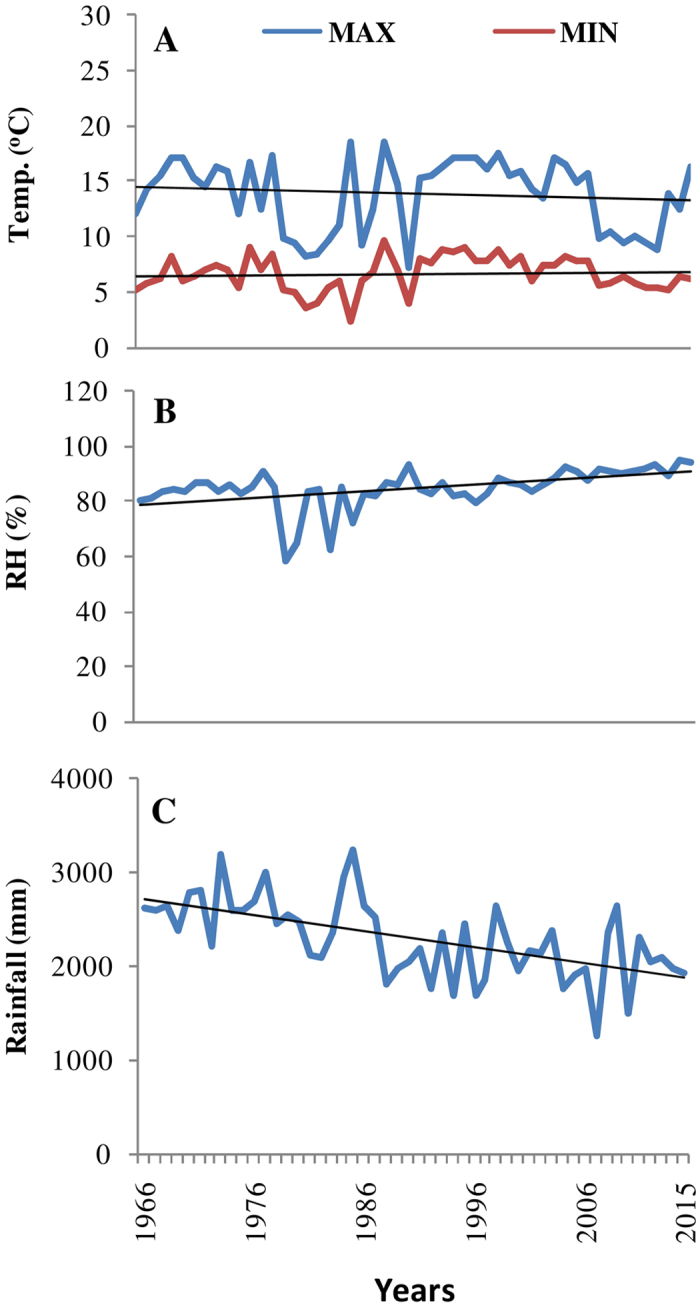
Climatic condition of the study area: (**A**) annual mean temperature (^o^C), (**B**) annual mean relative humidity (%) and (**C**) annual mean of rain fall (mm).

The meteorological data indicated significant change in climatic condition in the study area during the last five decades. The annual mean temperature registered abrupt changes over the years with mean maximum temperature below the trend line during 1970s, while above to the trend during 1980s–1990s. The mean maximum temperature showed a falling trend during the study period while mean minimum temperature showed a slight increasing trend (Fig. 1A). The seasonal temperature observation showed maximum change in winter temperature followed by monsoon while the summer temperature did not change much (Supplementary Fig. 1A–C). The mean annual relative humidity showed an increasing trend with about 10% increase at present in comparison to 1966 (Fig. 1B). The maximum change in mean relative humidity was in monsoon (45 to 94%) followed by summer and winter seasons (Supplementary Fig. 1D–F). In contrast, the mean precipitation decreased from 2500 to 1800 mm during the study period (Fig. 1C). The annual average temperature of India has registered more abrupt changes as compared to the global average during last century^48^. A rapid warmness in eastern Himalayan temperature has been reported during last five decades from other sources as well^17^,^49^. As observed in Darjeeling district, in the present study, however, a falling trend in annual mean temperature in north-eastern India has been reported earlier^48^. On the seasonal scale during 1901–2007, the maximum temperature has significantly increased in all the seasons while rainfall decreased in different part of the India^48^. The changes in the climatic conditions of the study area is also corroborated by the decreasing Emberger index^50^ in last half century (from 652 to 222) (Table 1).

### Level of inorganic and organic pollution

*Heterodermia diademata*, an epiphytic, foliose lichen grows on diversified substrate and good accumulator of atmospheric depositions due to presence of hair like structures, rhizines, present on the lower surface of the lichen. The species was selected for further study to compare the changes in climatic conditions during the study period and to trace the impact of global climate change. The epiphytic, foliose lichens such as *Parmelia caperata*, *Parmelia sulcata* and *Phaeophyscia hispidula* has been used extensively to monitor atmospheric depositions and to study air quality^27^,^31^.

**Figure 2:**
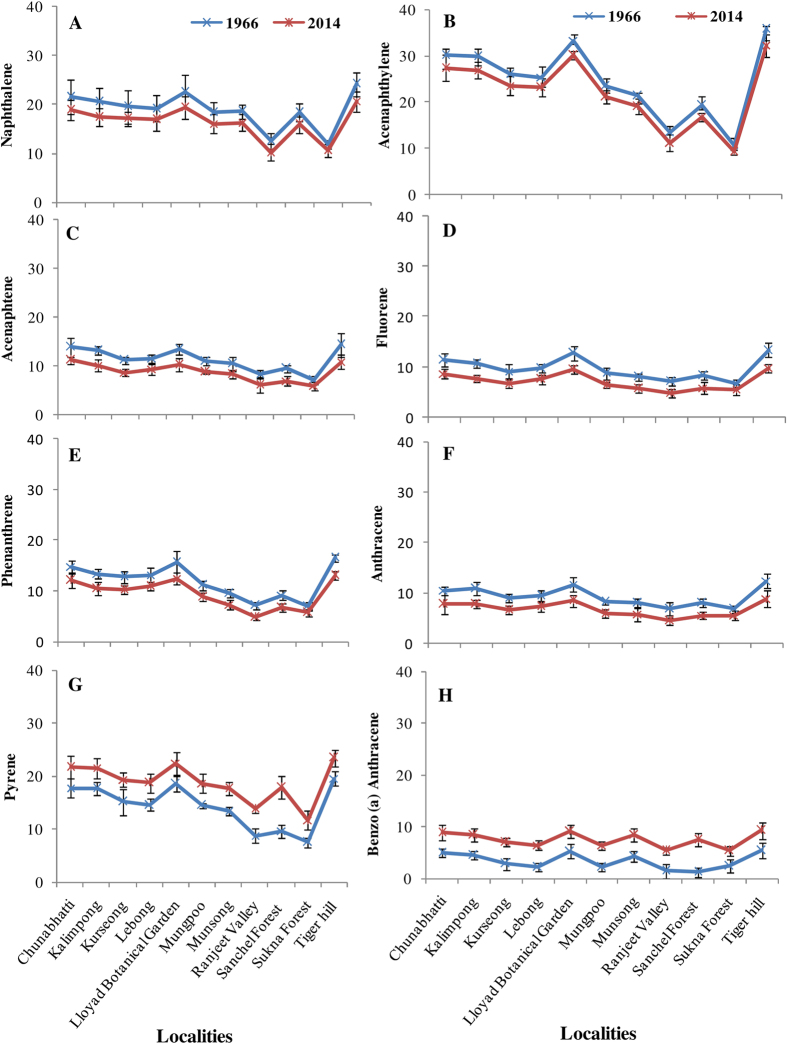
Level of polycyclic aromatic hydrocarbon (PAHs in μg g^−1^ dw) in herbarium and fresh samples of *H. diademata* (**A**) Naphthalene, (**B**) Acenaphthylene, (**C**) Acenaphtene, (**D**) Fluorene, (**E**) Phenanthrene, (**F**) Anthracene, (**G**) Pyrene, (**H**) Benzo(a)anthracene.

**Figure 3:**
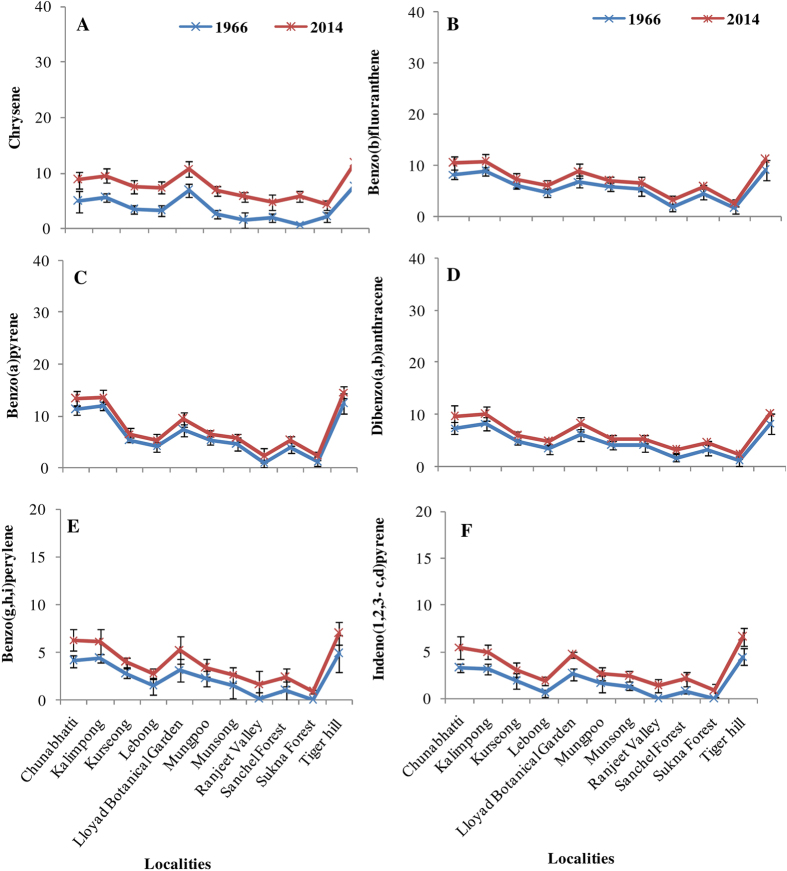
Level of polycyclic aromatic hydrocarbon (PAHs in μg g^−1^ dw) in herbarium and fresh samples of *H. diademata* (**A**) Chrysene, (**B**) Benzo(b)fluoranthene, (**C**) Benzo(a)pyrene, (**D**) Dibenzo(a,b)anthracene, (**E**) Benzo(g,h,i)perylene, (**F**) Indeno(1, 2, 3-c,d)pyrene.

The main sources of PAHs in environment are incomplete combustion of fossil fuel in industrial activities, power generation, vehicular emissions and forest fire^51^. A total of 14 PAHs were analysed and categorized as low molecular weight (LMPAH; containing ≤3 rings, m.w. < 200) and high molecular weight (HMPAH; containing ≥4 rings, m.w. > 200) (Supplementary Table 2). Interestingly, the HMPAHs showed a remarkable increase (1.2 to 2.2 fold) in the recent samples, while LMPAHs decreased (1.1 to 1.4 fold) in comparison to the past samples (Figs 2 and 3). However, the average level of LMPAHs was higher in comparison to HMPAHs in both the samples of past and present. Accumulation of PAHs also showed significant difference at various localities, but in contrast to metals (discussed below), the locality wise trend for various PAHs was almost similar in both past and present specimens. The highest level observed in the lichens collected from Tiger hill area, while the lichens collected from Sukhna forest and Ranjeet valley were generally lowest in PAHs accumulation (Figs 2 and 3). PAHs are semivolatile organic compounds and hydrophobic in nature thus exist both in the gaseous and particulate phase of the air. Lichens have been already recommended as good bioindicators of particulate phase PAHs^52^. Recently, Loppi *et al.*^53^ showed a significant correlation between the concentrations of specific PAH in lichens and in gas phase of air. The gas phase PAHs accumulate in the photobiont layer of the lichens where they have been reported to localize inside the cell and their concentration remained stable upon washing and rain^54^. Thus, lichens may, as well, be utilized as suitable monitors of gas phase PAHs^53^,^54^.

In the present study, the site wise variation in PAHs accumulation was more prominent for HMPAHs than the LMPAHs. This demonstrates that the HMPAHs were more confined to their source of emission while the LMPAHs were mobile and thus more dispersed in the study area^55^. Since the HMPAHs generally present in the particulate phase of air, tend to get deposited on various substrates^56^. Whereas, LMPAHs compounds exist mainly in gas-phase of atmosphere and can be influenced by the meteorological factors^52^. The extensive deforestation in the Himalayan region in the last decades might be a reason for decrease in LMPAHs due to changes in wind speed, direction and temperature. The contrast trend observed in the accumulation of HMPAHs and LMPAHs in the past and present samples showed that the preservation of lichens did not significantly affect the level of PAHs.

**Figure 4:**
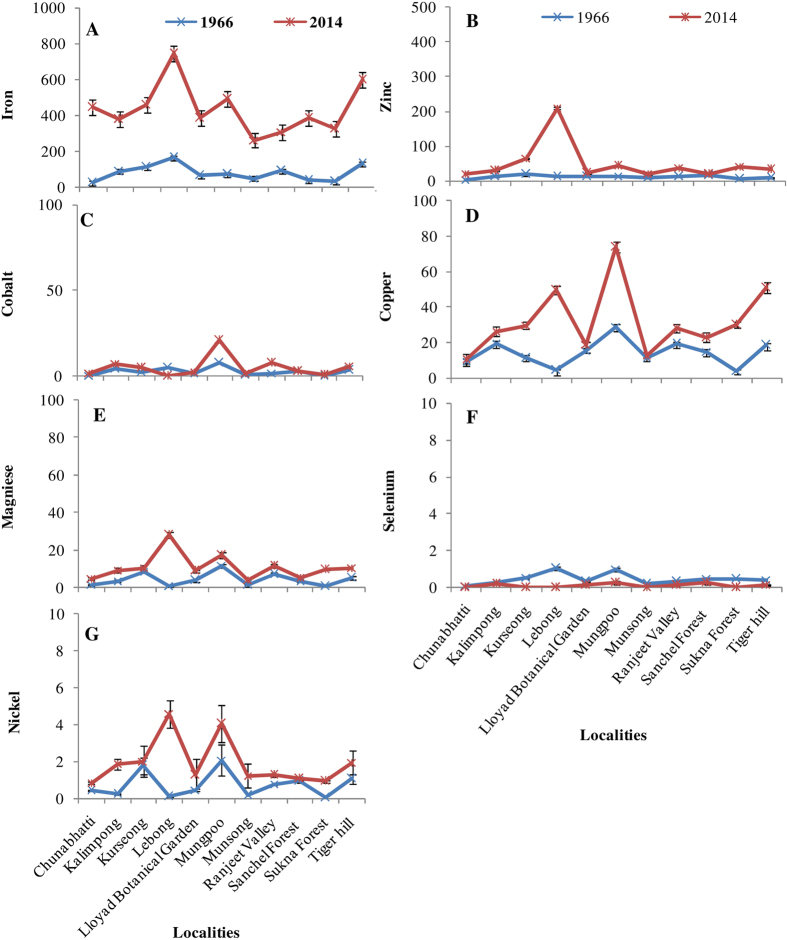
Elements accumulation (μg g^−1^ dw) in herbarium and fresh samples of *H. diademata* (**A**) Iron, (**B**) Zinc, (**C**) Cobalt, (**D**) Copper, (**E**) Manganese, (**F**) Selenium (**G**) Nickel.

**Figure 5:**
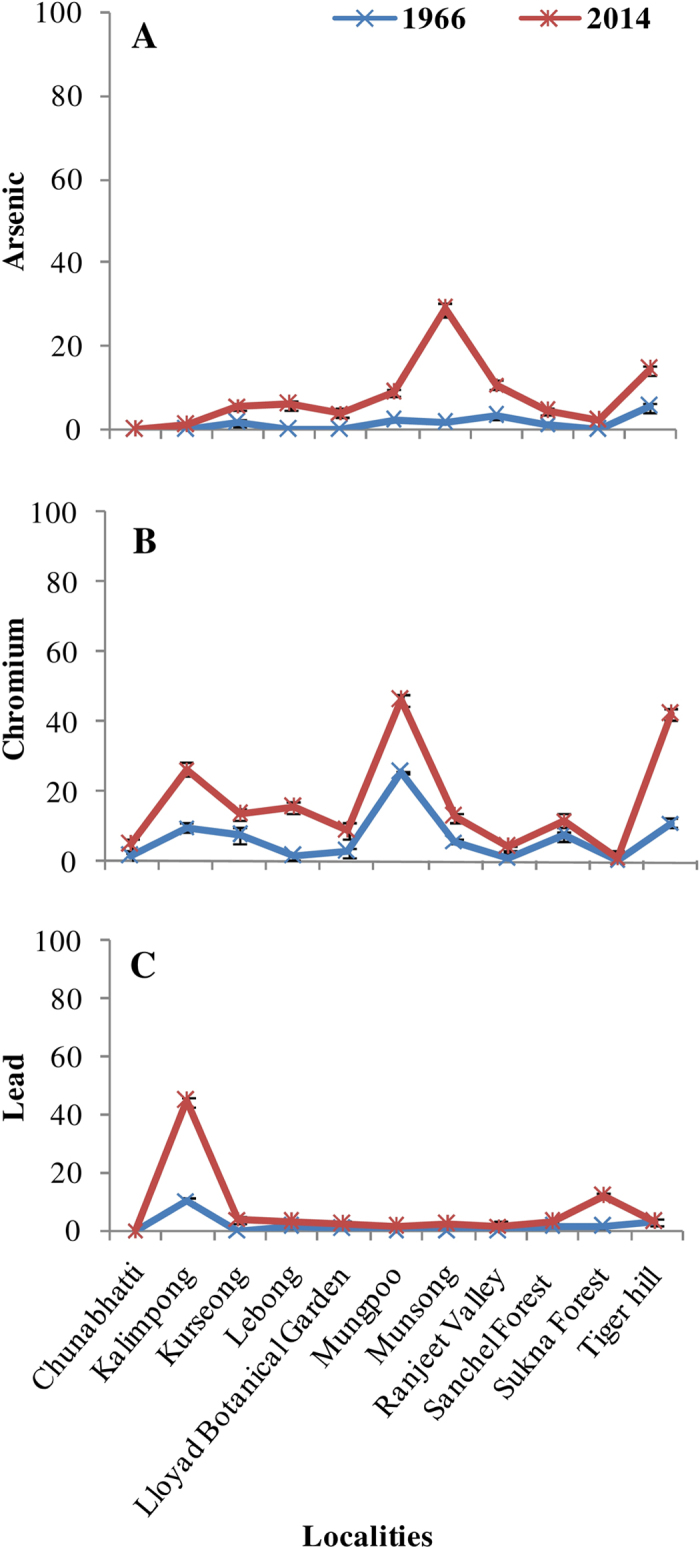
Elements accumulation (μg g^−1^ dw) in herbarium and fresh samples of *H. diademata* (**A**) Arsenic, (**B**) Chromium, (**C**) Lead.

In general, the recent specimens accumulated more elements with an average 2.5 to 5 fold increases in various elements compared to the past specimens (Supplementary Table 3). Copper and Zn were most abundant in both old and new lichen collections while selenium was least, and its accumulation further decreased in recent samples. In contrast, arsenic (As) accumulation was almost 7 fold higher in the recent specimens which may be a reason for reduced accumulation of Se in *H. diademata*. Such antagonistic response of As and Se has been observed in higher plants such as rice^46^. A significant site wise variation was observed in elements accumulation in both old and new lichen samples (Fig. 4). The samples from Mungpoo area showed higher accumulation of most of the elements followed by Lebong area, whereas least accumulation was observed from Chunabhatti followed by Sukhna forest area in both old and new lichen samples. Toxic elements like As, Cr and Pb showed high increase in the recent samples in comparison to the past samples with maximum concentration being in the samples collected from urban localities, such as Kalimpong, Munsong and Mungpoo (Fig. 5). The higher level of toxic elements indicate their source through urban activities, such as use of paints, preservatives, pesticides and coal and peat combustion for home heating^57^. Additionally, the ground water of Darjeeling has high probability of having As contamination, as the sulphides of the Darjeeling Himalayas contain up to 0.8% arsenic^58^, as well as the ground water of West Bengal and neighbouring North-eastern states are severely contaminated with As^59^,^60^. Thus, the increased dependency on ground water for irrigation and house hold purposes in the recent past might be the reason for elevated level of As in the study area. The probable reason for higher concentration of other elements may be due to the influence of traffic, deforestation and increased agricultural and anthropogenic activities in the study area^20^,^57^,^61^.

### Carbon, nitrogen concentration and carbon discrimination

**Figure 6:**
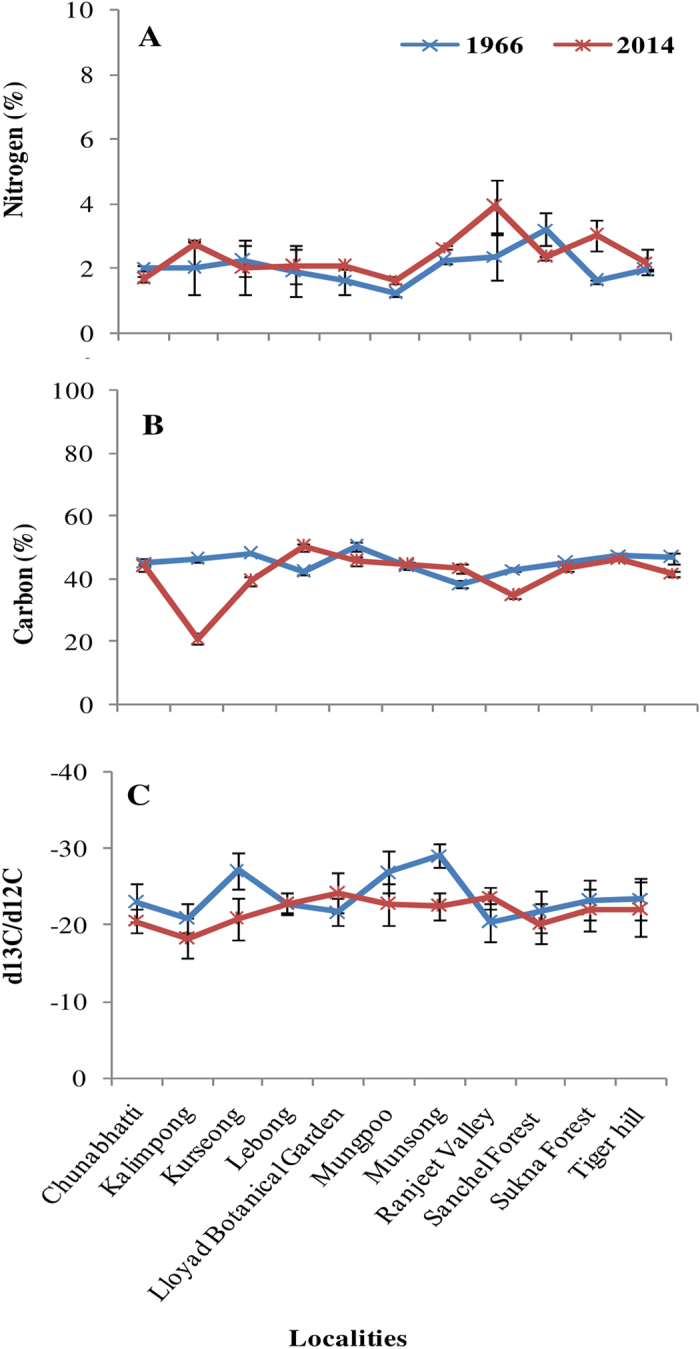
Level of (**A**) Nitrogen (%), (**B**) Carbon (%) and (**C**) Carbon isotope composition (δ^13^C) in the samples of *H. diademata* for 1966 and 2014.

In the present study, the concentration of N showed an average increase while C concentration decreased in the recent lichen samples in comparison to the old samples (Fig. 6A,B; Supplementary Table 3). The increase in N concentration indicates an increase in atmospheric N from various sources, such as use of fertilizers and urban emissions. The maximum N concentration was in the samples from Ranjeet Valley, an area used for tea farming, followed by the urban localities, Kalimpong and Munsong (Fig. 6A). The N concentration showed an inverse relationship with C concentration in the present study which indicates *H. diademata* a nitrogen sensitive species and probably could not maintain a balanced C to N stoichiometry between the symbiont partners during excess N^62^,^63^. Nitrogen in the form of NH_3_ has been reported to modify lichen chemistry and physiology^64^. Several other factors such as lack of regulation of N uptake and available form of N (e.g. NH_4_^+^ is more damaging) and limitation of other nutrient elements may also cause N toxicity in lichens^65^.

The carbon isotope composition (δ^13^C) was, an average, lower (more ^13^C depleted) in old collection (mean δ^13^C −23.7 ± 2.8) as compared to the recent lichen samples (mean δ^13^C −21.8 ± 1.7) showing less carbon discrimination in the recent specimens (Supplementary Table 3). The δ^13^C showed strong variability at different localities in both old and new specimens ranging from −20.91 to −29.07 in herbarium specimens and from −18.22 to −24.18 in recent lichen collection (Fig. 6C). In the past, the lichens collected from Munsong area showed minimum δ^13^C followed by Kurseong and Mungpoo and the maximum was from Ranjeet Valley area followed by Kalimpong, while in the new lichen collection it was minimum in Lloyd Botanical Garden followed by Ranjeet valley and maximum in the lichens growing at Kalimpong.

The δ^13^C in lichens can be correlated with various atmospheric parameters like CO_2_ concentration, humidity, temperature etc. Since lichens use the standard Rubisco for carboxylation, the main factors influencing their δ^13^C are the δ^13^C of atmospheric CO_2_ and diffusion resistance between atmosphere and carboxylation site^66^,^67^. Since the emission from fossil fuel combustion and biomass destruction is almost depleted in δ^13^C, therefore, a continuous increase in CO_2_ level and decrease in δ^13^C in the air has been observed which has been more drastic during the last five- six decades^1^,^5^,^36^,^68^. A positive correlation between δ^13^C of air CO_2_ and lichen thalli was observed in the lichen specimens collected during 1846 to 1989^68^. Thus, the use of lichens for global change studies has been suggested^20^,^28^,^29^,^30^,^69^. A decrease in δ^13^C in herbarium specimens of various plants was found during last century^36^. However, δ^13^C is a combined record of the various climatic and physiological factors that affects C assimilation and respiration. Thus, the physiological, morphological, and source effects may cause high heterogeneity in δ^13^C of the lichens. The locality wise variation in δ^13^C, in the present study, may be due to the differences in CO_2_ diffusion resistance factors such as water content and other factors influencing photosynthetic rate, such as temperature and light. Variation in growth rate, age and chemical composition of the lichen, due to variation in habitat and elevation may also cause differences in CO_2_ diffusion path and consequently altering the CO_2_ inside the lichen thallus. The growth rate of lichens has been shown to change up to two orders of magnitude from warmer and wetter Peninsula to cold and dry valleys of Antarctica^23^.

Further, the δ^13^C values of old specimens exhibited more locality wise variability than the recent specimens. This demonstrates that the natural factors (level of CO_2_ in source air, temperature, water, altitude, steepness) were directly affecting the δ^13^C in the past specimens while in the recent years the abrupt change in the environmental conditions as well as the increased level of pollutants, may have changed lichen physiology at all the localities, thus, less responsive to the natural factors. Peñuelas and Azcon-Bieto^5^ also found a decrease in δ^13^C in the leaves of C3 and C4 plants during the recent decades. Another possibility could be the deforestation which may influence δ^13^C of ambient air in the study area. Since lichens are more sensitive to the anthropogenic pollution such as, automobile exhaust, dust and heavy metals than the higher plants therefore the disturbed physiological responses may overshadow the effect of global climate change indications, such as increase in CO_2_ level, depletion of δ^13^C, particularly in the past half century.

### Change in the diversity and community structure of lichens

**Figure 7:**
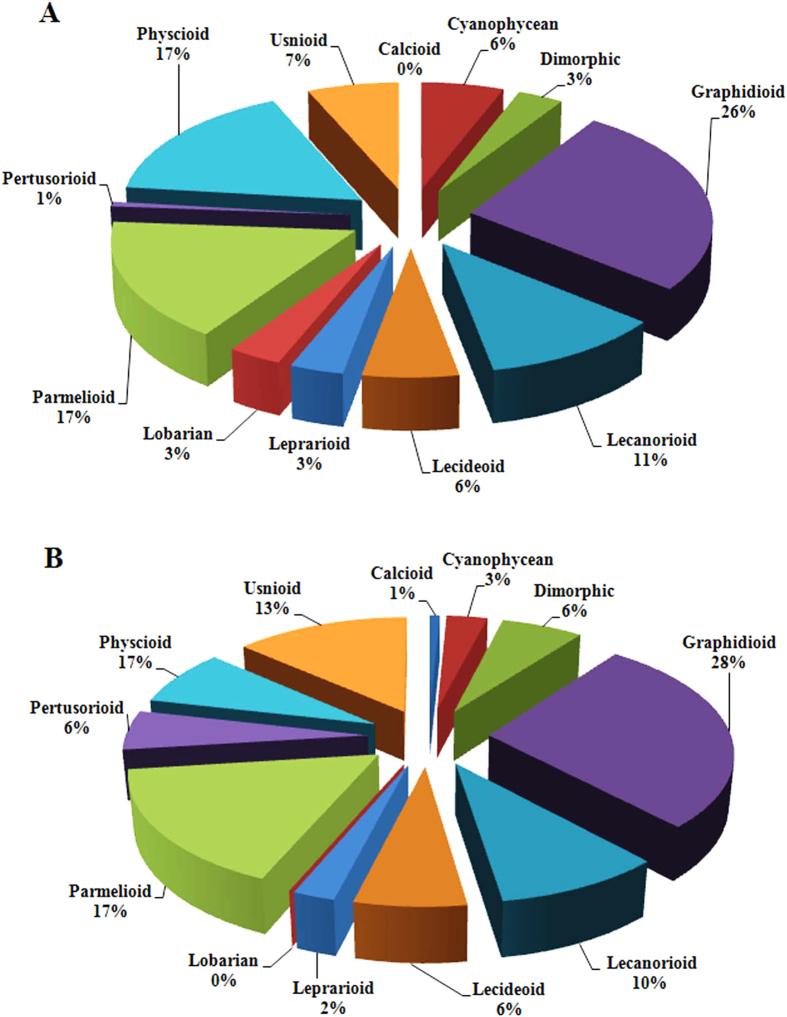
Shift in lichen community structure, (**A**) communities encountered during 1966 (**B**) communities encountered during 2014.

**Figure 8:**
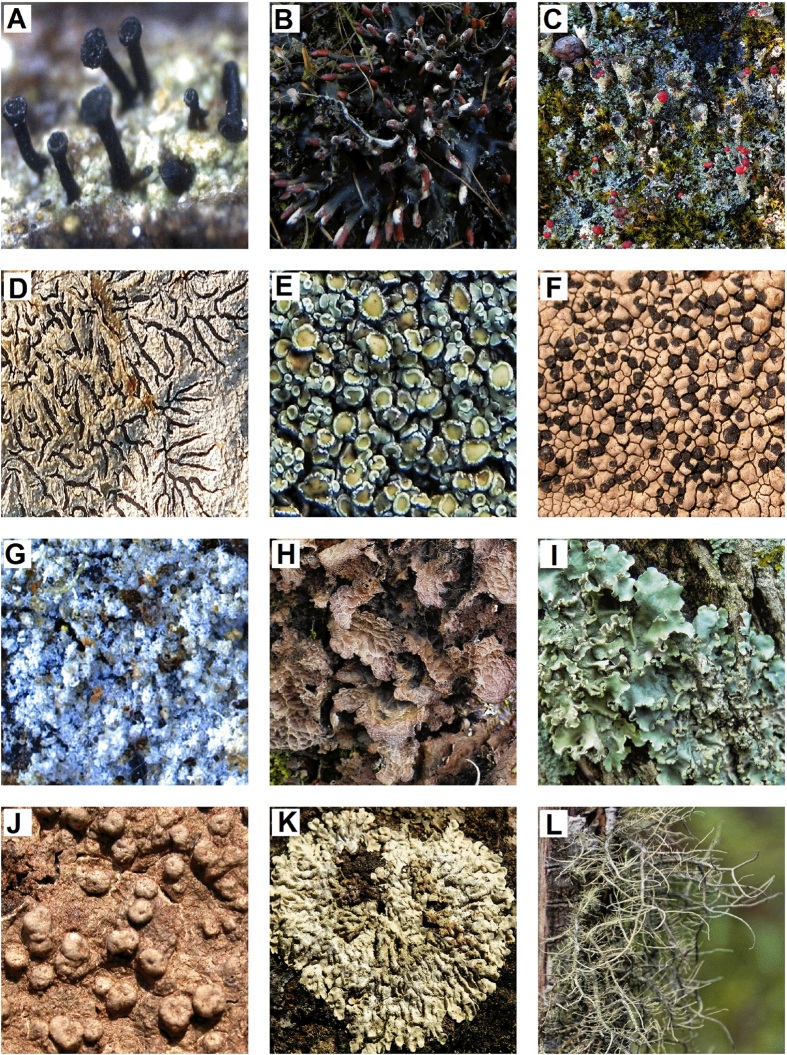
Lichen species representing 12 communities found in the study area: (**A**) Calcioid (**B**) Cyanophycean (**C**) Dimorphic (**D**) Graphidioid (**E**) Lecanorioid (**F**) Lecideoid (**G**) Leprarioid (**H**) Lobarian (**I**) Parmelioid (**J**) Pertusorioid (**K**) Physcioid (**L**) Usnioid.

A total of 251 species, belonging to 77 genera and 34 families of lichens represent the Darjeeling district of the state of West Bengal encompassing the herbarium records from 1966 and resent survey in 2014 (Supplementary Table 4). The total lichen species encountered in Darjeeling were belonging to 12 communities (Fig. 7). The species representing each community has been shown in Fig. 8. The herbarium collection documented 151 species of lichens belonging to 61 genera of 29 families from 11 localities^43^,^44^. The same localities were surveyed after a gap of 48 years and reported the occurrence of only 126 species belonging to 45 genera of 22 families, out of which only 26 species were common in both past and present study while 100 species found in the recent survey were entirely different from those found in 1966 (Supplementary Fig. 2A,B). The new survey revealed a significant change in growth form, habitat and community structure of lichens in the study area in comparison to herbarium record. Since the growth of various lichen species is largely dependent on the climate, a minor fluctuation in the climate may change the community structure^22^. Various studies have demonstrated that the changes in lichens community (increase or decrease in number of genus/species), and growth form with respect to global climate change^28^,^70^. In the present study, Graphidioid (26%), Parmelioid ≃ Physcioid (17%) and Lecanorioid (11%) were the major contributors in the past, while in the recent survey, Usnioid (13%) community almost doubled and Physcioid decreased to about half. Though, Graphidoid (28%) and Parmelioid (17%) communities did not change significantly. Among the minor communities, Dimorphic and Pertusorioid showed increase while Leprarioid and Cyanophycean showed decrease in the recent record (Fig. 2). Thus, in the recent samples a significant decrease in foliose lichens and increase in dimorphic lichens was observed. Interestingly, the Lobarian community showed complete absence while Calcioid was a new introduction in the study area in the recent survey. According to herbarium record, most of the lichens were inhibiting over bark and twigs (corticolous), whereas during present survey of study area majority of lichen species, particularly of Physcioid community, were growing over rocks (saxicolous). Earlier evidence also indicated that epiphytic species are increasing and terricolous species are declining due to global warming in Western Europe^70^. Isocrono *et al.*^34^ found a drastic change in lichen diversity in the city of Turin, north Italy during 1960 to 1996 with rapid disappearance and reappearance of various species. However, in the present study, the records of herbarium specimens between the study periods (1966 to 2014) were not available, thus possible disappearance and reappearance of one or more species during this period cannot be excluded. Therefore, the present study highlights the importance of periodic monitoring and maintenance of herbarium records.

Lichens often grow in extreme environments distributed from poles to the tropical regions and are good indicator of changing environmental conditions^23^. The change in lichen flora in the study area can be correlated with the change in climatic conditions and increased organic and inorganic pollutants in the last five decades, as evident by the increased accumulation of PAHs and metals/metalloid in *H. diademata*. Further, the Emberger Index showed drastic decrease pointing to the increasing dryness in the study area (Table 1). The change in moisture and precipitation has been reported to cause major change in growth rate and shift in lichen habitat^71^. Thus, the decline in communities of lichens associated with cyanobacteria, such as Cyanophycean and Lobarian may be related to the decrease in the precipitation, as the cyanobacterial symbionts are often found in cool, wet and nitrogen limited conditions^72^,^73^,^74^,^75^. The Lobarian are also sensitive to pollutants such as nitrogen^76^ although in the present study, the level of N did not show increase at the locality (Kurseong) from where they were reported in the past (Supplementary Table 4). In contrast to cyanobacterial symbionts, the species associated with green-algal symbionts are generally more frequent in drier climate^74^. The Usneioid community has registered increase in the recent survey, although, interestingly most of the species belonging to this community were confined to specific localities, in contrast to the more dispersed in the past (Supplementary Table 4). Further, the past collection was dominated by *Usnea* and *Everniastrum* while in the recent survey *Everniastrum* decreased drastically and the number of *Usnea* and *Ramalina* increased. The other green-algal containing lichens, such as Pertusorioid, Calcioid and Dimorphic also increased except the Physcioid community. The decrease in Physcioid could be related to deforestation and increased urbanization in Kalimpong and Munsong, the prime localities of this community in the past.

Though the contribution of Parmelioid community did not change significantly, the number of species belonging to this community decreased in the recent survey. The reduction in members of Parmelioid community can be correlated with increased anthropogenic activities such as, vehicular emissions and construction works leading to deposition of dust and heavy metals on the surface of lichen thallus^20^,^61^. The most frequent species of lichen family Graphidiaceae, Pyrenulaceae, and Lecanoraceae apparently showed higher resistance to pollution than the less frequent ones in the study area. Thus, the drastic shift observed in the lichen community structure might be a combined result of change in the climatic conditions, such as temperature and precipitation, as well as the habitat destruction through harvesting of trees for commercial motives and land uses, such as for agricultural, housing and road construction purposes^77^. The lack of correlation between δ^13^C of lichen samples and δ^13^C of air in the present study, indicates that the short term studies (≤5 decades) including herbarium specimens of lichens, might reflect recent micro-environmental conditions obscuring the changes in atmospheric CO_2_ concentration and ^13^C composition.

## Conclusion

From the present study it can be concluded that the level of trace elements and PAHs, particularly high molecular weight PAHs has substantially increased in Darjeeling district in last five decades, which reflect rapid urbanization in the area. The study highlights the importance of herbarium specimens for reconstructing historical trend in atmospheric deposition and to study the temporal variation in the community structure of lichens. The shift in lichen community structure in response to climatic conditions and environmental pollutants warrant its use as a model to study the global climate and/or local microclimate change. The lack of information between the studied time periods emphasize the importance of sample collection, curation and conservation of herbarium records for understanding long term changes and more complete information about the vulnerability of species.

## Additional Information

**How to cite this article**: Bajpai, R. *et al.* Change in atmospheric deposition during last half century and its impact on lichen community structure in Eastern Himalaya. *Sci. Rep.* **6**, 30838; doi: 10.1038/srep30838 (2016).

## Supplementary Material

Supplementary Information

## Figures and Tables

**Table 1:** Emberger Index showing change in climatic conditions in Darjeeling (Eastern Himalaya).

Time period	Emberger Index (EI)
1966–1975	652.53 (±123)
1976–1985	257.45 (±83)
1986–1995	253.23 (±57.6)
1996–2005	244.55 (±77)
2006–2015	222.36 (±59)

The Emberger’s index is calculated as follows: EI = 100 × P/T_max_^2^ − T_min_^2^, where P is average annual precipitation, T_max_ is average maximum temperature of warmest month, T_min_ is average minimum temperature of coldest month. The values are mean of 10 years.
