# Supplementary material for: Change in atmospheric deposition during last half century and its impact on lichen community structure in Eastern Himalaya
Source: Sci Rep. 2016 Aug 9;6:30838. doi: 10.1038/srep30838 (PMC4977567; doi:10.1038/srep30838)
Supplement: Supplementary Information [file srep30838-s1.pdf]

Supplementary Material

Change in atmospheric deposition during last half century and its impact on lichen community structure in Eastern Himalaya

Rajesh Bajpai*[,1], Seema Mishra*[,2], Sanjay Dwivedi[2] & Dalip Kumar Upreti[#,1]

[1]Lichenology Laboratory, Plant Diversity Systematics and Herbarium Division

[2]Plant Ecology and Environment Science Division

CSIR-National Botanical Research Institute, Rana Pratap Marg, Lucknow - 226001, India

*These authors contributed equally

[#]Corresponding Author:

Dalip Kumar Upreti

Chief Scientist

Lichenology Laboratory

Plant Diversity Systematics and Herbarium Division

CSIR-National Botanical Research Institute

Rana Pratap Marg, Lucknow-226001, Uttar Pradesh, India

Phone: ++91-522-2297850, Fax: ++91-522-2205836, 220583639

Email: upretidknbri@gmail.com

Supplementary Table 1: Details of sample collection sites re-visited in 2014 according to the herbarium records of 1966 of Darjeeling, West Bengal.

S. No.	Localities	Coordinates	Altitude (mt)	Details of sites
1.	Chunabhatti	N26º50′45.03″ E88º20′14.05″	772	Site situated along with road side
2.	Kalimpong	N27º00′01.02″ E88º15′34.05″	2203	Urban area
3.	Kurseong	N26º54′21.02″ E88º17′30.05″	1627	Semi Urban area
4.	Lebong	N27º03′08.05″ E88º16′31.04″	1911	State roadside area
5.	Lloyad Botanical Garden	N27º02′37.04″ E88º15′43.77″	2000	Centre of the city
6.	Mungpoo	N26º58′12.08″ E88º21′26.01″	1451	Semi Urban area
7.	Munsong	N26º09′08.07″ E88º05′23.03″	1500	Semi urban area, road side
8.	Ranjeet Valley	N27º03′07.07″ E88º16′25.03″	1871	Tea garden surrounded by closer canopy of forest trees
9.	Sanchal Forest	N27º00′17.07″ E88º15′43.05″	2269	Road side but, close canopy of trees away from urban area
10.	Sukna forest	N26º48′01.02″ E88º21′26.04″	242	Dense forest patches away from road side
11.	Tiger Hill	N26º59′53.06″ E88º16′21.04″	2491	Tourist spot with frequent vehicular activities and scattered forest patches

Supplementary Table 2: Mean and range of PAHs (µg g^{-1}dw) in samples of *H. diademata* together with indicates individual PAHs concentration for both the sampling period

	PAHs	1966	2014
**PAH ≤ 3 rings**	Naphthalene<br>(2-rings, M.W. 178.17)	**18.86**^{b}<br>(12.6-24.2)	**16.33**^{a}<br>(10.2-20.7)
	Acenaphthylene<br>(3-rings, M.W. 152.19)	**24.47**^{b}<br>(10.6-35.8)	**21.94**^{a}<br>(9.4-32.3)
	Acenaphtene<br>(3-rings, M.W. 154.08)	**11.33**^{b}<br>(7.2-14.4)	**8.80**^{a}<br>(6.0-11.2)
	Fluorene<br>(3-rings, M.W. 166.22)	**9.70**^{b}<br>(6.8-13.4)	**7.17**^{a}<br>(4.8-9.9)
	Phenanthrene<br>(3-rings, M.W. 178.23)	**11.86**^{b}<br>(6.9-16.6)	**9.33**^{a}<br>(4.8-13.1)
	Anthracene<br>(3-rings, M.W. 178.23)	**9.28**^{b}<br>(6.8-12.4)	**6.75**^{a}<br>(4.5-8.9)
**PAH ≥ 4 rings**	Pyrene<br>(4-rings, M.W. 202.25)	**14.31**^{a}<br>(7.5-19.6)	**18.84**^{b}<br>(11.7-23.5)
	Benzo(a)anthracene<br>(4-rings, M.W. 228.28)	**3.40**^{a}<br>(1.3-5.5)	**7.54**^{b}<br>(5.4-9.4)
	Chrysene<br>(4-rings, M.W. 228.29)	**3.69**^{a}<br>(0.7-7.8)	**7.55**^{b}<br>(4.2-11.7)
	Benzo(b)fluoranthene<br>(5-rings, M.W. 252.30)	**5.72**^{a}<br>(1.6-9.1)	**7.27**^{b}<br>(2.7-11.2)
	Benzo(a)pyrene<br>(5-rings, M.W. 252.32)	**6.22**^{a}<br>(0.9-12.5)	**7.77**^{b}<br>(2.4-14.6)
	Dibenzo(a,b)anthracene<br>(5-rings, M.W. 278.34)	**4.86**^{a}<br>(1.2-8.4)	**6.40**^{b}<br>(2.3-10.5)
	Benzo(g,h,i)perylene<br>(6-rings, M.W. 276.33)	**2.23**^{a}<br>(0.0-4.9)	**3.88**^{b}<br>(0.9-7.0)
	Indeno(1,2,3- c,d)pyrene<br>(6-rings, M.W. 276.33)	**1.78**^{a}<br>(0.0-4.5)	**3.30**^{b}<br>(0.9-6.6)

BDL: Blow detection limit; values presented in brackets are the range of particular PAHs and bold values represent mean. All values are mean of three replicates±S.D. ANOVA significant at p≤0.01. Small letters (a and b) indicate significantly different values between years (DMRT, p≤0.05).

Supplementary Table 3: Mean and range of elements concentration (μg g^{-1}dw), nitrogen, carbon and carbon isotope composition ($\delta^{13}C$) in *H. diademata* for both the sampling period.

Elements	1966	2014
Cobalt (Co)	**2.57**[a] (BDL-7.47)	**5.08**[b] (BDL-21.03)
Copper (Cu)	**14.25** [a] (4.07-29.00)	**32.24** [b] (10.85-73.85
Manganese (Mn)	**4.48** [a] (0.69-11.83)	**11.02** [b] (4.93-28.21)
Nickel (Ni)	**0.74** [a] (0.05-2.09)	**1.96** [b] (0.86-4.60)
Iron (Fe)	**79.91** [a] (26.45-167)	**435.10** [b] (308-744)
Selenium (Se)	**0.46** [b] (0.10-1.05)	**0.09** [a] (BDL-0.26)
Zinc (Zn)	**10.95** [a] (3.95-17.81)	**49.08** [b] (18.01-209)
Chromium (Cr)	**6.82** [a] (0.47-25.53)	**17.09** [b] (1.33-46.15)
Arsenic (As)	**1.33** [a] (BDL-5.24)	**7.77** [b] (BDL-28.79)
Lead (Pb)	**1.54** [a] (BDL-10.36)	**6.76** (BDL-44.36)
Carbon (%C)	**45.26** (38.57-50.45)	**41.39** [a] (21.28-50.18)
Nitrogen (%N)	**2.06** [a] (1.26-3.23)	**2.41** [b] (1.67-3.93)
Discrimination 13C/12C	**-23.67** (-20.39- (-)29.07)	**-21.77** (-18.22- (-)24.18)

BDL: Blow detection limit; values presented in brackets are the range of particular metals and bold values represent mean. All values are mean of three replicates±S.D. ANOVA significant at p≤0.01. Small letters (a and b) indicate significantly different values between years (DMRT, p≤0.05).

Supplementary Table 4: Diversity and distribution of lichen taxa growing at various localities of Darjeeling, West Bengal.

S.No.	Lichen Species	Family	1966's											2014's											Common
			1	2	3	4	5	6	7	8	9	10	11	1	2	3	4	5	6	7	8	9	10	11	
1.	*Anthracothecium himalayense* (Räsänen) Awas.	Pyrenulaceae				●																			
2.	*Anthracothecium macrosporum* (Hepp.) Müll. Arg.	Pyrenulaceae														●			●			●			
3.	*Anthracothecium manipurense* Müll. Arg.	Pyrenulaceae				●																			
4.	*Anthracothecium prasinum* (Eschw.) R.C. Harris	Pyrenulaceae														●			●						
5.	*Arthonia antiliarum* (Fee) Nyl.	Arthoniaceae															●								
6.	*Arthonia* sp.	Arthoniaceae															●								
7.	*Arthonia subvelata* Nyl.	Arthoniaceae										●													
8.	*Arthonia tumidula* (Ach.) Ach.	Arthoniaceae															●								
9.	*Arthothelium dispersum* (DC) Mudd	Arthoniales										●													
10.	*Bacidia alutacea* (Kremelh.) Zahlbr.	Ramalinaceae													●		●								
11.	*Bacidia laurocerasi* (Delise ex Dube) Ozenda & Claiz	Ramalinaceae																					●		
12.	*Bacidia phaeolomoides* (Müll. Arg.) Zahlbr.	Ramalinaceae										●													
13.	*Bacidia polychroa* (Th. Fr.) Körb.	Ramalinaceae										●													
14.	*Bacidia rubella* (Hoffm.) Mass.	Ramalinaceae														●			●			●			
15.	*Bacidia submedialis* (Nyl.) Zahlbr.	Ramalinaceae														●									
16.	*Bacidina delicata* (Larbel. Ex Leight) V. Wirth & Vezda	Ramalinaceae																	●						
17.	*Bacidina phacodes* (Körb.) Vì zda	Ramalinaceae										●													
18.	*Bapalmuia buchananii* (Stirt.) Kalb & Lücking	Pilocarpaceae											●												
19.	*Bryoria himalayana* (Mont.) Brodo & Hawksw.	Parmeliaceae												●											
20.	*Buellia alboatrior* (Nyl.) Zahlbr.	Caliciaceae									●														
21.	*Bulbothrix isidiza* (Nyl.) Hale	Parmeliaceae				●			●		●		●			●		●			●			●	●
22.	*Bulbothrix meizospora* (Nyl.) Hale	Parmeliaceae		●		●						●	●												
23.	*Bulbothrix setschwanensis* (Zahlbr) Hale	Parmeliaceae																			●	●			
24.	*Bulbothrix tabacina* (Mont. & Bosch) Hale	Parmeliaceae																		●					
25.	*Candelariella aurella* (Hoffm.) Zahlbr	Candelariaceae												●											

26.	*Canoparmelia pustulencens* (Kurok.) Elix	Parmeliaceae														●										
27.	*Catillaria leptocheiloides* (Nyl.) Zahlbr	Catillariaceae										●														
28.	*Cetrelia braunsiana* (Müll. Arg.) W. Culb. & C. Culb.	Parmeliaceae	●					●																		
29.	*Cetrelia cetrarioides* (Del. Ex. Dubey) W. Culb. & C. Culb	Parmeliaceae	●					●																		
30.	*Cetrelia olivetorum* (Nyl.) Culb. & C. Culb.	Parmeliaceae						●																		
31.	*Cetrelia pseudolivetorum* (Asah) W. Culb. & C. Culb.	Parmeliaceae				●		●																		
32.	*Chaenotheca brunneola* (Ach.) Müll. Arg.	Coniocybaceae				●																				
33.	*Chiodecton leptosporum* Müll. Arg.	Roccellaceae			●	●		●				●					●			●			●			●
34.	*Cladonia awasthiana* Ahti & Upreti	Cladoniaceae																						●		
35.	*Cladonia cartilaginea* Müll. Arg.	Cladoniaceae													●									●		
36.	*Cladonia chlorophaea* (Florke ex Sommerf.) Spreng.	Cladoniaceae										●														
37.	*Cladonia coniocraea* (Florke) Spreng.	Cladoniaceae												●												
38.	*Cladonia crispata* (Ach.) Flotow.	Cladoniaceae																					●			
39.	*Cladonia fruticulosa* Kremp.	Cladoniaceae															●		●							
40.	*Cladonia furcata* (Huds.) Schrad.	Cladoniaceae	●	●		●								●		●		●						●		●
41.	*Cladonia yunnana* (Vain.) Abbayes ex Bay & Jiang	Cladoniaceae												●												
42.	*Coccocarpia palmicola* (Spreng.) Arv. & D.J. Galloway	Coccocarpaceae				●			●	●	●															
43.	*Collema nigrescens* (Huds.) DC	Collemataceae		●								●														
44.	*Collema subflaccidum* Degel.	Collemataceae				●																				
45.	*Collema subnigrescens* Degel.	Collemataceae							●																	
46.	*Cratiria obscurior* (Stirt.) Marbach & Kalb.	Caliciaceae										●														
47.	*Cryptothecia awasthii* Makh. & Patw.	Arthoniaceae																				●	●			
48.	*Cryptothecia candida* (Krempel.) R. Sant.	Arthoniaceae											●													
49.	*Cryptothecia conferta* A. L. Smith	Arthoniaceae									●															
50.	*Cryptothecia effusa* (Müll. Arg.) R. Sant.	Arthoniaceae											●													
51.	*Cryptothecia stirtonii* A. L. Smith	Arthoniaceae							●																	
52.	*Cryptothecia subtecta* Stirt.	Arthoniaceae										●	●													

53.	*Dibaeis baeomyces* (L.f.) Rambold & Hertel	Baeomycetaceae												●												
54.	*Diorygma heiroglyphicum* (Pers.) Steiger & Kalb.	Graphidaceae														●	●		●	●		●				
55.	*Diorygma junghuhnii* (Mont. & Bosch.) Kalb, Staigu & Elix	Graphidaceae				●			●												●	●	●			●
56.	*Diorygma megasporum* Kalb, Staiger & Elix	Graphidaceae																				●				
57.	*Diorygma soozanum* (Zahlbr) M. Nakan. & Kashiw.	Graphidaceae																					●			
58.	*Diorygma tuberculosum* (Stirt.) Kalb. & al.	Graphidaceae			●					●																
59.	*Diploschistes muscorum* (Scop.) R. Sant.	Thelotremataceae		●																						
60.	*Diploschistes scruposus* (Scherb.) Norm.	Thelotremataceae		●																						
61.	*Dirinaria aegialita* (Afz. In Ach.) Moore	Physciaceae															●					●				
62.	*Dirinaria applanata* (Fée) D. D. Awasthi	Physciaceae										●														
63.	*Dirinaria consimilis* (Stirt.) D. D. Awasthi	Physciaceae									●															
64.	*Dirinaria picta* (Sw.) Clem. & Shaer.	Physciaceae										●														
65.	*Enterodictyon indicum* Müll. Arg.	Roccellaceae										●														
66.	*Evernia mesomorpha* Nyl.	Parmeliaceae													●											
67.	*Everniastrum cirrhatum* (Fr.) Hale	Parmeliaceae	●	●	●	●		●				●					●	●	●	●						●
68.	*Everniastrum nepalense* (Taylor) Hale ex Sipman	Parmeliaceae				●						●	●													
69.	*Everniastrum vexans* (Zahlbr.) Hale ex Sipman	Parmeliaceae										●	●													
70.	*Graphidastra byssiseda* (Müll. Arg.) G. Thor	Roccellaceae				●		●					●													
71.	*Graphina aeola* (Stirt.) Zahlbr.	Graphidaceae		●																						
72.	*Graphina canaliculata* (Fée) Müll. Arg.	Graphidaceae		●					●																	
73.	*Graphina gracilis* (Fr.) Müll. Arg.	Graphidaceae										●														
74.	*Graphina poitaei* (Fée) Müll. Arg.	Graphidaceae								●		●														
75.	*Graphina spilota* (Stirt.) Zahlbr.	Graphidaceae										●														
76.	*Graphis aurita* Eschw. In Mart.	Graphidaceae															●									
77.	*Graphis caesiella* Vain.	Graphidaceae																●								
78.	*Graphis chloroalba* Makhija & Adaw	Graphidaceae																●								
79.	*Graphis chlorotica* A. Massal	Graphidaceae												●		●							●			

80.	*Graphis crebra* Vain.	Graphidaceae														●										
81.	*Graphis dendrogramma* Nyl.	Graphidaceae																	●			●				
82.	*Graphis dimidiata* Vain.	Graphidaceae																	●							
83.	*Graphis duplicata* Ach.	Graphidaceae				●																				
84.	*Graphis enteroleuca* (Ach.) Luck.	Graphidaceae																	●							
85.	*Graphis handelii* Zahlbr.	Graphidaceae								●																
86.	*Graphis illinata* Eschw.	Graphidaceae							●																	
87.	*Graphis implexula* Stirt.	Graphidaceae									●															
88.	*Graphis lineola* Ach.	Graphidaceae								●																
89.	*Graphis longiramea* Müll. Arg.	Graphidaceae						●																		
90.	*Graphis nuda* (Magn.) Staiger & Lucking	Graphidaceae																					●			
91.	*Graphis pinicola* Zahlbr.	Graphidaceae												●												
92.	*Graphis platycarpa* Eschw.	Graphidaceae			●																					
93.	*Graphis proserpens* Vain.	Graphidaceae												●												
94.	*Graphis rimulosa* (Mont.) Trevis.	Graphidaceae						●																		
95.	*Graphis scripta* (L.) Ach.	Graphidaceae	●		●									●		●				●			●			●
96.	*Graphis tenella* Ach.	Graphidaceae																				●				
97.	*Graphis verminosa* Müll. Arg.	Graphidaceae		●				●																		
98.	*Haematomma leprarioides* (Vain.) Vain.	Haematommataceae										●	●													
99.	*Haematomma puniceum* (Sw.) Massal	Haematommataceae				●			●			●								●			●			●
100.	*Haematomma wattii* (Stirt.) Zahlbr.	Haematommataceae				●																				
101.	*Hemithecium divaricoides* (Räsänen) Tewari & Upreti	Graphidaceae														●	●									
102.	*Hemithecium pyrrhochroa* (Mont. et v.d. Bosch) V. Tewari & Upreti	Graphidaceae														●			●							
103.	*Herpothallon australasicum* (Elix) Elix & G. Thor	Arthoniaceae																			●					
104.	*Heterodermia awasthii* (Kurok.) D.D. Awasthi	Physciaceae											●													
105.	*Heterodermia boryi* (Fee) Singh & Sinha	Physciaceae													●											
106.	*Heterodermia comosa* (Eschw.) Follman & Redon	Physciaceae														●	●									

107.	*Heterodermia diademata* (Taylor) D.D. Awasthi	Physciaceae	●	●	●	●	●	●	●	●	●	●	●	●	●	●	●	●	●	●	●	●	●	●	●
108.	*Heterodermia dissecta* (Kurok.) D.D. Awasthi	Physciaceae		●		●																			
109.	*Heterodermia firmula* (Nyl.) Trevis.	Physciaceae				●																			
110.	*Heterodermia flabellata* (Fée) D.D. Awasthi	Physciaceae										●													
111.	*Heterodermia himalayensis* (D.D. Awasthi) D.D. Awasthi	Physciaceae									●														
112.	*Heterodermia hypoleuca* (Ach.) Trevis.	Physciaceae													●	●							●		
113.	*Heterodermia incana* (Stirt.) D.D. Awasthi	Physciaceae										●	●												
114.	*Heterodermia isidiophora* (Vain.) D.D. Awasthi	Physciaceae				●	●					●					●	●							●
115.	*Heterodermia japonica* (Sato.) Swinsc. & Krog	Physciaceae				●						●					●								●
116.	*Heterodermia leucomelos* (L.) Poelt	Physciaceae				●																			
117.	*Heterodermia obscurata* (Nyl.) Trevis.	Physciaceae										●	●												
118.	*Heterodermia pseudospeciosa* (Kurok.) W. Culb.	Physciaceae				●						●	●										●	●	●
119.	*Heterodermia punctifera* (Kurok.) D.D. Awasthi	Physciaceae											●												
120.	*Heterodermia rubescens* (Räsanen) D.D. Awasthi	Physciaceae										●													
121.	*Heterodermia rubricosa* (Stirt.) Poelt	Physciaceae				●																			
122.	*Heterodermia speciosa* (Wulf.) Trevis	Physciaceae														●									
123.	*Heterodermia tremulans* (Müll. Arg.) W.L. Culb.	Physciaceae							●																
124.	*Hypotrachyna physcioides* (Nyl.) Hale	Parmeliaceae				●																			
125.	*Hypotrachyna adducta* (Nyl.) Hale	Parmeliaceae										●	●									●		●	●
126.	*Hypotrachyna awasthii* Hale & Patwardhan	Parmeliaceae															●		●	●			●		
127.	*Hypotrachyna crenata* (Kurok.) Hale	Parmeliaceae														●	●			●					
128.	*Hypotrachyna exsecta* (Taylor) Hale	Parmeliaceae													●	●				●					
129.	*Hypotrachyna flexilis* (Kurok.) Hale	Parmeliaceae			●					●	●									●	●				●
130.	*Hypotrachyna imbricatula* (Zahlbr) Hale	Parmeliaceae												●		●			●	●					
131.	*Hypotrachyna incognita* (Kurok.) Hale	Parmeliaceae																		●					
132.	*Hypotrachyna infirma* (Kurok.) Hale	Parmeliaceae			●	●						●	●	●	●	●					●		●		●
133.	*Hypotrachyna koyaensis* (Asahina) Hale	Parmeliaceae		●	●	●										●	●			●					●

134.	*Hypotrachyna minarum* (Vain.) Korg & Swinscow	Parmeliaceae																●							
135.	*Hypotrachyna osseoalba* (Vain.) Y.S. Park and Hale	Parmeliaceae	●	●	●			●					●	●	●										●
136.	*Hypotrachyna sublaevugata* (Nyl.) Hale	Parmeliaceae																		●	●				
137.	*Lasallia pustulata* (L.) Merat	Umbilicariaceae														●									
138.	*Laureraindica* Upreti & A. Singh	Trypetheliaceae								●															
139.	*Lecanora achroa* Nyl.	Lecanoraceae																			●				
140.	*Lecanora allophana* (Ach.) Nyl.	Lecanoraceae									●														
141.	*Lecanora chlarotera* Nyl.	Lecanoraceae														●									
142.	*Lecanora concilians* Nyl.	Lecanoraceae														●									
143.	*Lecanora frimbriatula* Stirt.	Lecanoraceae													●	●									
144.	*Lecanora helva* Stizenb.	Lecanoraceae													●										
145.	*Lecanora interjecta* Müll. Arg.	Lecanoraceae											●												
146.	*Lecanora tropica* Zahlbr.	Lecanoraceae													●										
147.	*Lecidea granifera* (Ach.) Vain.	Lecideaceae						●			●	●									●	●			●
148.	*Lecidella euphora* (Florke) Hertel	Lecanoraceae													●										
149.	*Leptogium azureum* (Sw.) Mont.	Collemataceae		●	●																				
150.	*Leptogium cochleatum* (Dicks.) P.M. J¯rg. & P. James	Collemataceae							●	●															
151.	*Leptogium corticola* (Taylor) Tuck	Collemataceae																	●						
152.	*Leptogium cyanescens* (Rabenh.) Korb.	Collemataceae				●		●	●	●								●	●	●					●
153.	*Leptogium denticulatum* Nyl.	Collemataceae											●												
154.	*Leptogium javanicum* Mont.	Collemataceae			●																				
155.	*Leptogium moluccanum* (Pers.) Vain.	Collemataceae			●																				
156.	*Leptogium trichophorum* Müll. Arg.	Collemataceae	●		●																				
157.	*Letrouitia domingensis* (Pers.) Hafellner & Bellem	Letrouitiaceae									●														
158.	*Lobaria isidiosa* (Müll. Arg.) Vain.	Lobariaceae			●																				
159.	*Lopadium pezizoideum* (Ach.) Korb.	Ectolechiaceae			●																				
160.	*Mycobilimbia hunana* (Zahlbr.) D.D. Awasthi	Lecanorales										●													

161.	*Mycoblastus indicus* D. D. Awasthi & M.R. Agarwal	Mycoblastaceae				●						●	●											
162.	*Myelochroa sikkimensis* Divakar, Sinha, Upreti & Elix	Parmeliaceae														●								
163.	*Myelochroa subaurulenta* (Nyl.) Elix & Hale	Parmeliaceae				●						●	●											
164.	*Myelochroa xantholepis* (Mont. & Bosch) Elix & Hale	Parmeliaceae				●																		
165.	*Ocellularia emersa* (Kremp.) Mull. Arg.	Thelotremataceae												●										
166.	*Opegrapha prosodea* Ach.	Roccellaceae				●			●															
167.	*Opegrapha rufescens* Pers.	Roccellaceae							●															
168.	*Opegrapha subvulgata* Nyl.	Roccellaceae							●															
169.	*Opegrapha vulgata* Ach.	Roccellaceae															●							
170.	*Parmelaria thomsonii* (Stirt.) D.D. Awasthi	Parmeliaceae		●		●				●														
171.	*Parmelinella wallichiana* (Taylor) Elix & Hale	Parmeliaceae		●		●		●			●		●				●		●			●		●
172.	*Parmotremahahabianum* (Gyeln.) Hale	Parmeliaceae				●																		
173.	*Parmotrema praesorediosum* (Nyl.) Hale	Parmeliaceae				●																		
174.	*Parmotrema pseudonilgherrense* (Asahina) Hale	Parmeliaceae		●		●																		
175.	*Parmotrema cristiferum* (Taylor) Hale	Parmeliaceae							●															
176.	*Parmotrema reticulatum* (Taylor) Choisy	Parmeliaceae	●		●	●	●	●	●			●			●		●	●	●	●	●			●
177.	*Parmotrema sancti-angelii* (Lynge) Hale	Parmeliaceae				●						●	●											
178.	*Parmotrema tinctorum* (Nyl.) Hale	Parmeliaceae			●		●			●	●	●	●				●			●	●			●
179.	*Parmotrema ultralucens* (Körg.) Hale	Parmeliaceae				●						●	●											
180.	*Peltigera dolichorriza* (Nyl.) Nyl.	Peltigeraceae				●																		
181.	*Peltigera polydactyla* (Neck.) Hoffm.	Peltigeraceae				●																		
182.	*Peltigera pruinosa* Gyeln.	Peltigeraceae				●			●															
183.	*Pertusaria leucosora* Nyl.	Pertusariaceae																					●	
184.	*Pertusaria leucosorodes* Nyl.	Pertusariaceae												●		●				●			●	
185.	*Pertusaria melastomella* Nyl.	Pertusariaceae																					●	
186.	*Pertusaria pertusa* (L.) Tuck.	Pertusariaceae									●													
187.	*Pertusaria quassiae* Fée	Pertusariaceae																				●	●	

188.	*Pertusaria subrigida* Müll. Arg.	Pertusariaceae													●							
189.	*Pertusaria tuberculifera* Nyl.	Pertusariaceae																				●
190.	*Phaeographis angulosa* Müll. Arg.	Graphidaceae																	●			●
191.	*Phaeographis megistocarpa* (Leight.) Müll. Arg.	Graphidaceae			●																	
192.	*Phaeographis formula* (Stirt.) V. Tewari & Upreti	Graphidaceae													●							
193.	*Phaeographis submarcescens* (Leight.) Zahlbr.	Graphidaceae			●						●											
194.	*Phaeophyscia endococcinia* Körb.	Physciaceae	●																			
195.	*Phyllopsora corallina* (Eschw.) Müll. Arg.	Ramalinaceae																			●	
196.	*Phlyctis karnatakana* S. Joshi & Upreti	Thelotremataceae																				●
197.	*Phlyctis subhimalayensis* Joshi & Upreti	Thelotremataceae													●			●				
198.	*Physcia crispa* Nyl.	Physciaceae																		●		
199.	*Platygramme caesiopruinosa* (Fée) Fée.	Graphidaceae								●												
200.	*Polyblastia cupularia* A. Massal	Porinaceae								●												
201.	*Porina tetracerae* (Afz.) Müll. Arg.	Porinaceae								●												
202.	*Porina subcutanea* Ach.	Porinaceae																●		●	●	
203.	*Pseudopyrenula pupula* (Ach.) Müll. Arg.	Trypetheliaceae			●																	
204.	*Pyrenula aggregata* (Fèe) Fèe	Pyrenulaceae			●																	
205.	*Pyrenula cuyabensis* (Malme) R.C. Harris	Pyrenulaceae																	●			
206.	*Pyrenula interducta* (Nyl.) Zahl.	Pyrenulaceae	●																			
207.	*Pyrenula leucotrypa* (Nyl.) Upreti,	Pyrenulaceae										●										
208.	*Pyrenula macrospora* (Degel.) Coppins & James	Pyrenulaceae													●	●						●
209.	*Pyrenula marvalensis* Vain.	Pyrenulaceae													●						●	
210.	*Pyrenula mastophoroides* (Nyl.) Zahlbr.	Pyrenulaceae													●							
211.	*Pyrenula nitida* (Weig.) Ach.	Pyrenulaceae							●													
212.	*Pyrenula nodulata* (Stirt.) Zahl.	Pyrenulaceae									●											
213.	*Pyrenula pyrenuloides* (Mont.) R.C. Harris	Pyrenulaceae													●							
214.	*Pyrenula scutata* (Stirt.) Zahl.	Pyrenulaceae						●	●													

215.	*Pyrenula subducta* (Nyl.) Müll. Arg.	Pyrenulaceae											●													
216.	*Pyrenula subindica* Upreti	Pyrenulaceae				●																				
217.	*Pyrenula sublaevigata* (Pat. & Makhija) Upreti	Pyrenulaceae																					●			
218.	*Pyxine cocoes* (Sw.) Nyl.	Physciaceae								●																
219.	*Pyxine phillippina* Vain.	Physciaceae									●															
220.	*Pyxine retirugella* Nyl.	Physciaceae									●															
221.	*Pyxine sorediata* (Ach.) Mont.	Physciaceae		●		●																				
222.	*Ramalina conduplicans* Vain.	Ramalinaceae	●		●	●				●		●	●		●						●		●	●		●
223.	*Ramalina hossei* Vain.	Ramalinaceae										●				●										
224.	*Ramalina roesleri* (Hochst.) Hue	Ramalinaceae	●	●	●				●						●	●					●					●
225.	*Rinodina conradii* Körbör	Physciaceae				●																				
226.	*Solorina simensis* Hochst.	Peltigeraceae												●												
227.	*Stereocaulon foliolosum* Nyl.	Stereocaulaceae	●			●									●			●								●
228.	*Stereocaulon massartianum* Hue.	Stereocaulaceae												●			●									
229.	*Stereocaulon piluliferum* Th. Fr.	Stereocaulaceae				●																				
230.	*Sticta weigelii* (Ach.) Vain.	Lobariaceae				●																				
231.	*Tephromela khatiensis* (Räsänen) Lumbsch	Tephromelataceae																					●			
232.	*Thecaria quassiicola* Fée	Graphidaceae				●																				
233.	*Tomasellia cinchonarum* Müll. Arg.	Trypetheliaceae	●			●																				
234.	*Tomasellia himalayensis* D. D. Awasthi & Agarwal	Trypetheliaceae	●			●						●														
235.	*Trapelia coarctata* (Sm. & Sow.) Choisy	Tapeliariaceae												●												
236.	*Trypethelium assimile* Stirt.	Trypetheliaceae										●														
237.	*Trypethelium indutum* Stirt.	Trypetheliaceae										●														
238.	*Tylophoron moderatum* Nyl.	Lecanorales				●			●			●	●													
239.	*Usnea aciculifera* Vain.	Parmeliaceae														●							●			
240.	*Usnea baileyi* (Stirt.) Zahlbr.	Parmeliaceae			●	●	●										●	●					●			●
241.	*Usnea bismolliscula* Zahlbr.	Parmeliaceae												●		●		●					●			

242.	*Usnea cineraria* Mot.	Parmeliaceae							●				
243.	*Usnea compressa* Taylor in Hook.f.	Parmeliaceae		●		●	●						
244.	*Usnea dendritica* Stirt.	Parmeliaceae							●				
245.	*Usnea eumitrioides* Mot.	Parmeliaceae	●			●	●				●	●	●
246.	*Usnea galbinifera* Asahina	Parmeliaceae								●			
247.	*Usnea leucospilodea* Nyl.	Parmeliaceae									●		
248.	*Usnea luridorufa* Stirt.	Parmeliaceae									●		
249.	*Usnea pangiana* Stirt.	Parmeliaceae							●				
250.	*Usnea rubicunda* Stirt.	Parmeliaceae			●	●	●						
251.	*Usnea undulata* Stirt.	Parmeliaceae						●			●		

1, Tiger Hill; 2, Sanchel Forest; 3, Mungpoo; 4, Kurseong; 5, Lloyad Botanical Garden; 6, Lebong; 7, Ranjeet Valley; 8, Sukna Forest; 9, Chunabhatti; 10, Kalimpong; 11, Munsong

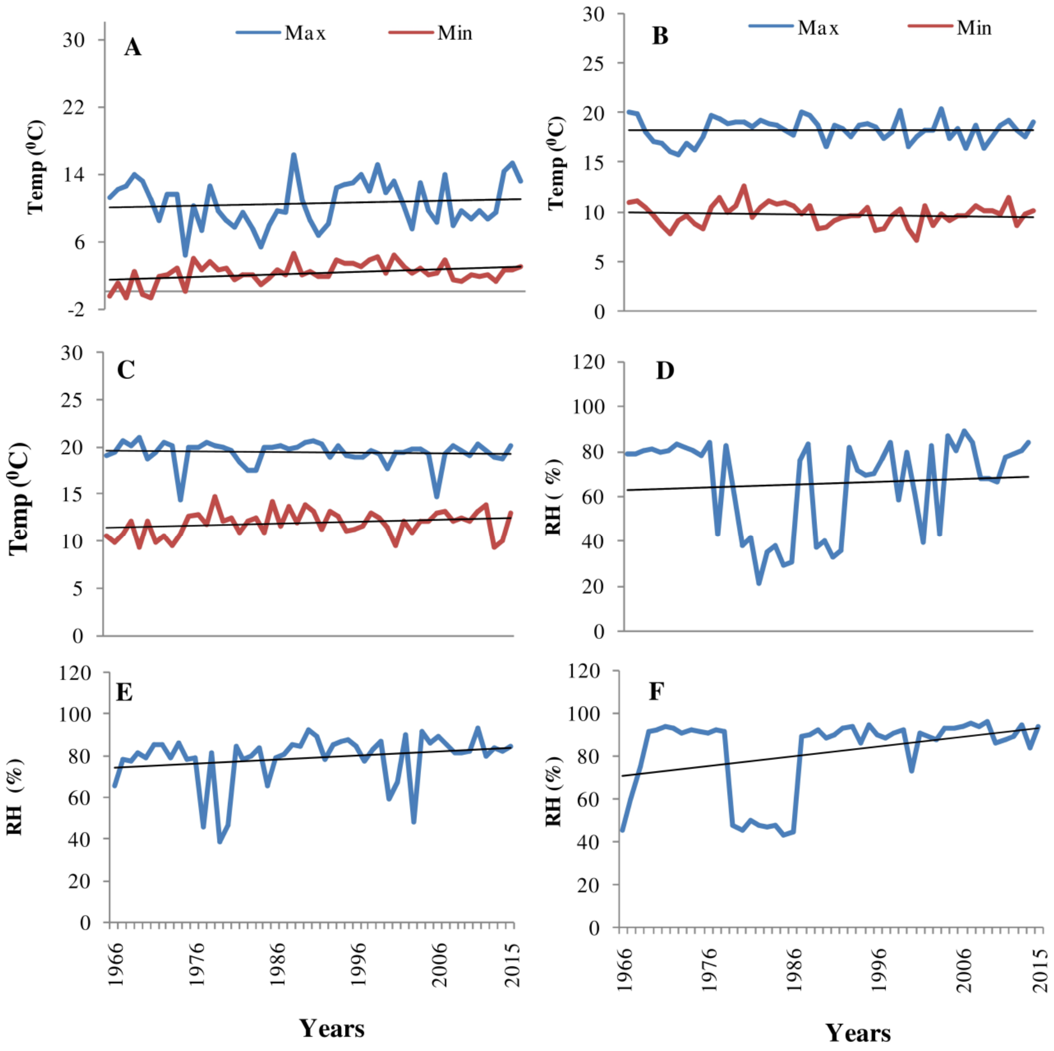

Supplementary Figure 1: Annual mean seasonal variations in the temperature (ºC) and relative humidity (%) of the study area: **(A)** Winter temperature, **(B)** Summer temperature, **(C)** Monsoon temperature, **(D)** Winter relative humidity, **(E)** Summer relative humidity, **(F)** Monsoon temperature.

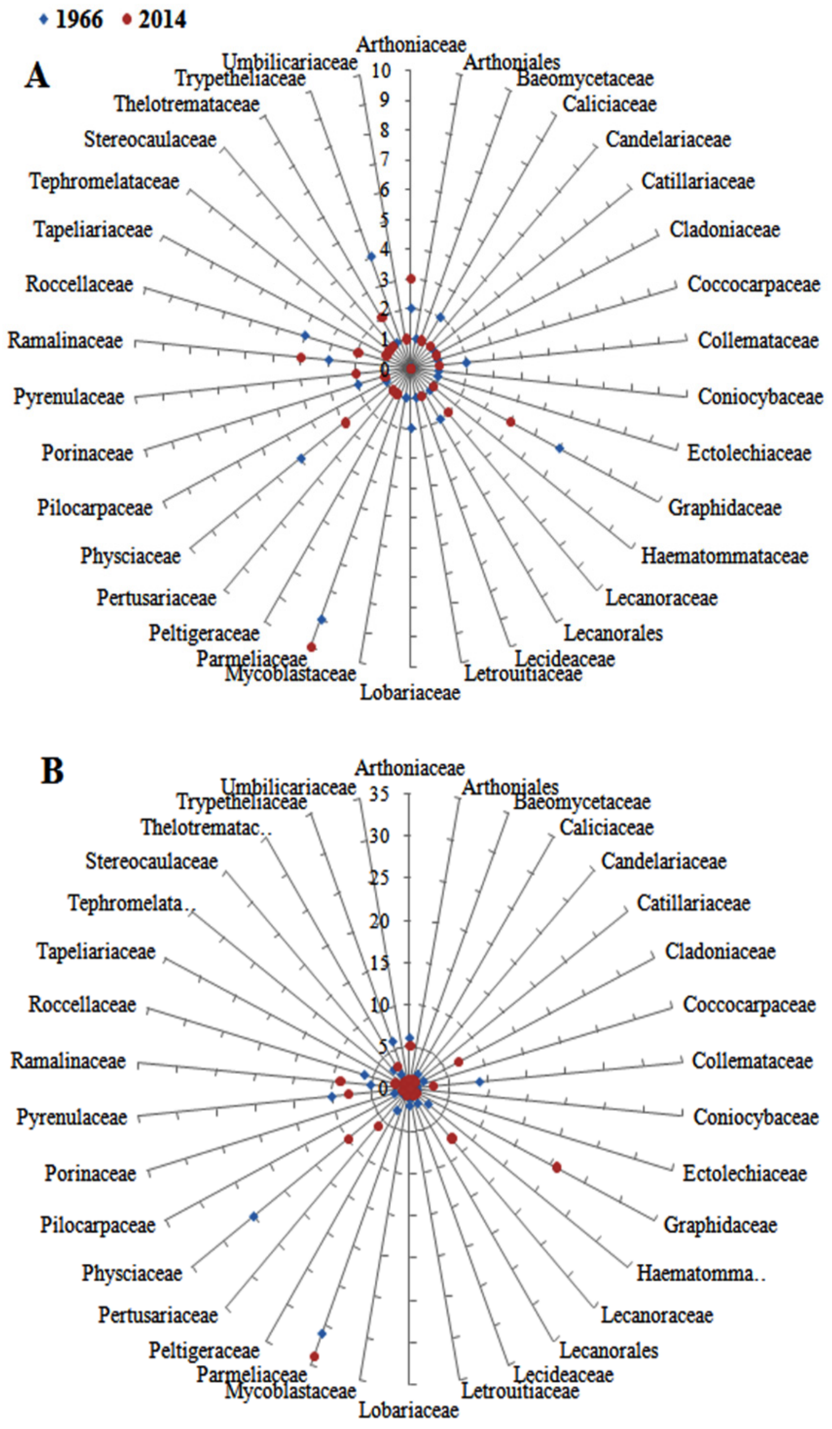

Supplementary Figure 2: Past and present lichen diversity, **A.** Families representing number of genera, **B.** Families representing number of species.
